# Resolution of 20-year refractory hiccups with ultrasound-guided phrenic nerve radiofrequency modulation and bilateral stellate ganglion block: a case report

**DOI:** 10.3389/fphys.2026.1622330

**Published:** 2026-03-04

**Authors:** Fan Lu, Ting Wang, Ling Ye

**Affiliations:** 1 Department of Pain Management, West China Hospital, Sichuan University, Chengdu, China; 2 Department of Anesthesia & Operation Center, West China Hospital, Sichuan University, Chengdu, China

**Keywords:** phrenic nerve block, radiofrequency modulation, refractory hiccups, stellate ganglion block, ultrasound-guided

## Abstract

Chronic refractory hiccups significantly impair quality of life, especially with prolonged symptom duration. This case highlights the therapeutic potential of a combined interventional approach in managing chronic refractory hiccups, particularly in patients with prolonged symptom duration unresponsive to conventional treatments. A 52-year-old man presented with a 20-year history of persistent, debilitating hiccups, often triggered after meals and resistant to pharmacological therapy. His symptoms had worsened in recent years, occasionally accompanied by reflux and vomiting, with minimal relief from anti-reflux medications and neural supplements. Further evaluation revealed pathological acid reflux and excessive supra-gastric belching, suggesting gastroesophageal reflux disease (GERD) as a contributing factor. After comprehensive assessment and informed consent, the patient underwent a series of ultrasound-guided interventions, including bilateral stellate ganglion blocks (SGBs) and phrenic nerve blocks. Although initial symptom relief was achieved, hiccups partially recurred, prompting the use of pulsed radiofrequency modulation (PNRF) of the phrenic nerve in combination with targeted analgesic injections. Following treatment, the patient experienced a significant and sustained reduction in hiccup frequency and severity, with only occasional brief relapses. Continued follow-up and additional sessions of phrenic nerve modulation resulted in further symptom control. This case underscores the effectiveness of integrating sympathetic and somatic nerve modulation, particularly ultrasound-guided SGB and PNRF, in cases of intractable hiccups. Such a multimodal, image-guided strategy may offer meaningful relief for patients suffering from chronic, treatment-resistant hiccups and demonstrates the value of personalized, interventional pain management in complex functional disorders.

## Introduction

Hiccups, or “singultus,” are involuntary, repetitive spasms of the diaphragm and intercostal muscles, often accompanied by abrupt glottal closure, producing the characteristic “hic” sound (Chang and Lu, 2012; Rouse and Wodziak, 2018). Hiccups are classified by duration into three categories: transient hiccups, which last less than 48 h; persistent hiccups, which last more than 48 h but less than 1 month; and intractable hiccups, which persist for over a month (Moonen et al., 2022; Ramachandran, 2025). While transient hiccups are common and typically benign, persistent and intractable hiccups are rare and can lead to significant impairment in quality of life. Intractable hiccups are associated with fatigue, weight loss, and psychosocial distress, particularly in cases where symptoms persist over prolonged periods or resist standard therapeutic approaches (Cole and Plewa, 2023). The estimated population prevalence of chronic hiccups approximates 0.1%, while reaching 3%–10% in patients with gastroesophageal reflux disease (GERD) and showing similarly elevated rates in neurological disorders (Steger et al., 2015).

The pathophysiology of hiccups involves complex interactions between the central and peripheral nervous systems, particularly the phrenic, vagus, and sympathetic nerves, all of which influence diaphragmatic activity (Polito and Fellows, 2017). Current management algorithms for intractable hiccups recommend initial trial of non-pharmacological physical maneuvers before pharmacological intervention. Evidence-supported techniques include respiratory cycle interruption, nasopharyngeal stimulation, or diaphragmatic repositioning (Moretto et al., 2013; Smith and Busracamwongs, 2003). When these measures prove insufficient, pharmacotherapy typically employs proton pump inhibitors, antiemetics, or antipsychotics, though their efficacy in chronic cases remains variable and often transient (Jeon et al., 2018). This therapeutic limitation has spurred growing interest in targeted nerve blockade as a potentially more definitive intervention for refractory cases.

Phrenic nerve blocks have been proposed as a viable treatment option for intractable hiccups, with several studies demonstrating their safety and efficacy in alleviating symptoms (Edinoff et al., 2021; Gong et al., 2021). Ultrasound-guided phrenic nerve blocks allow precise targeting of the nerve involved in diaphragmatic spasms, potentially offering long-lasting symptom relief. Similarly, stellate ganglion blocks (SGBs) have been reported to modulate sympathetic nervous system activity, which may contribute to further improvement in patients with persistent hiccups (Zhong et al., 2023). Importantly, intravenous (IV) lidocaine has also been reported as an effective intervention for persistent hiccups, with several case reports demonstrating rapid resolution following IV administration (Coh et al., 2001; Dunst et al., 1993; Boulouffe and Vanpee, 2007). However, to date, there is limited literature on effective therapeutic strategies for managing chronic, refractory hiccups, particularly in cases with symptom durations exceeding 2 decades. This case report presents a unique instance of successful management of intractable hiccups with a 20-year disease history using a dual-modality treatment approach.

## Case presentation

A 52-year-old male with a 20-year history of intractable hiccups was referred to the pain management department for further evaluation. The patient’s hiccups occurred 2–3 times per week, with each episode lasting 10–30 min, typically subsiding during sleep. However, in the past 5 years, the frequency and severity of his hiccups worsened, particularly in the morning and after meals. These episodes were sometimes accompanied by acid reflux and vomiting.

Initial gastroenterology consultations revealed chronic non-atrophic gastritis and multiple A1-stage duodenal ulcers. Treatment included esomeprazole (40 mg/day; proton pump inhibito), colloidal bismuth pectin (450 mg/day; mucosal protectant), mecobalamin (1.5 mg/day; neurotrophic agent), paroxetine (20 mg/day; selective serotonin reuptake inhibitor), and estazolam (1 mg/day; benzodiazepine sedative), leading to initial symptom relief. Despite treatment, the patient’s hiccups persisted and worsened. In August 2024, his hiccups became daily and unrelenting, no longer ceasing during sleep. Esophageal pH and impedance monitoring revealed excessive supragastric belching and high-level pathological acid reflux, consistent with GERD and motility issues. MRI scans of the neck, chest, and abdomen were unremarkable. Pharmacological treatment with vonoprazan (20 mg/day; potassium-competitive acid blocker) and mosapride (15 mg/day; prokinetic agent) failed to provide significant improvement. Multidisciplinary consultation involving gastroenterology, psychiatry, pain management, and radiology led to a revised treatment plan that included pregabalin (300 mg/day; gabapentinoid neuromodulator), olanzapine (2.5 mg/day; atypical antipsychotic), paroxetine (20 mg/day), and vonoprazan (20 mg/day). These changes resulted in partial symptomatic relief, with some reduction in hiccup intensity but no meaningful decrease in daily frequency.

Given the refractory nature of his symptoms, the patient was transferred to the pain management department for interventional treatment. After obtaining ethical approval and informed consent, ultrasound-guided bilateral phrenic nerve blocks were performed on days one and two. Under ultrasound guidance, the phrenic nerve was targeted at the C6 level between the anterior scalene and sternocleidomastoid muscles. A 22-gauge needle was advanced in-plane, and 3 mL of 1% lidocaine was injected per side. Following the block, hiccups changed from continuous episodes to complete hiccup cessation lasting approximately 6 h, followed by recurrence later the same day. He subsequently underwent sessions of ultrasound-guided bilateral SGBs on five consecutive days, with one session per day. Under ultrasound guidance, a 22-gauge needle was advanced to the level of the C6 anterior tubercle, and 4 mL of 1% lidocaine was injected per side. Successful blockade was confirmed by the development of ipsilateral Horner’s syndrome. Bilateral procedures were performed sequentially with an interval of approximately 4 h, and no adverse events were observed. With completion of five SGB sessions, the hiccup frequency improved to three to four brief episodes per day, each lasting 10–30 min. After completion of the five SGB sessions and prior to escalation to radiofrequency treatment, the patient received a continuous intravenous lidocaine infusion (5 mg/kg over 2 h) under continuous cardiac and hemodynamic monitoring. No clinically significant changes in heart rate, blood pressure, or oxygen saturation were observed. This intervention produced mild but transient reduction in hiccup intensity. The patient was subsequently discharged with outpatient follow-up and reassessment planned.

**FIGURE 1:**
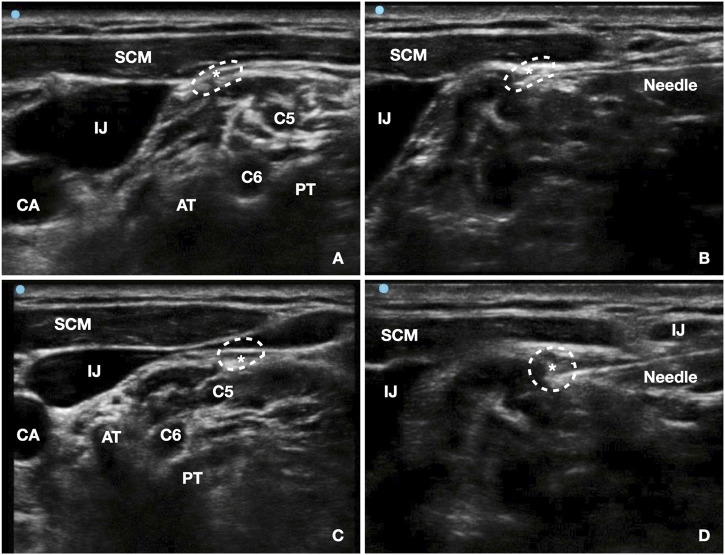
Ultrasound-guided bilateral phrenic nerve pulsed radiofrequency (PNRF) treatment. **(A)** Right-side PNRF localization view; **(B)** Right-side in-plane needle placement view; **(C)** Left-side PNRF localization view; **(D)** Left-side in-plane needle placement view. AT: anterior tubercle; PT: posterior tubercle; CA: carotid artery; IJ: internal jugular vein; SCM: sternocleidomastoid muscle; C5/C6: corresponding cervical nerve roots; *phrenic nerve indicated by white dotted circle.

At the 1-month follow-up after completion of the SGBs and lidocaine infusion, the patient continued to experience persistent symptoms, characterized by hiccup episodes occurring 2–3 times per day and lasting approximately 30 min. Given the ongoing symptom burden, the patient was readmitted for bilateral ultrasound-guided PNRF. Under ultrasound guidance, the phrenic nerve was targeted at the C6-C7 level between the anterior scalene and sternocleidomastoid muscles (Figure 1). The procedure involved 0.4V stimulation to induce diaphragmatic contraction, followed by radiofrequency modulation at 42 °C, 45V for 15 min. A 3 mL analgesic solution (2.5 mL ropivacaine, 2.5 mL lidocaine, 0.5 mL dexamethasone, 0.5 mL mecobalamin, and 4 mL saline) was injected. Within 24 h after PNRF combined with nerve block, hiccups completely resolved on the day of the procedure, similar to the transient effect observed after the initial phrenic nerve block. However, during the subsequent week, hiccup episodes fluctuated, occurring 2–3 times per day, although the duration of each episode was markedly reduced to 5–10 min. Over the following weeks, symptoms gradually stabilized. At 1 month post-PNRF, hiccups occurred 1–2 times per day with reduced severity. At 2 months post-PNRF, episodes further decreased to 0–2 brief events per day lasting approximately 5 min. By the third month, hiccups had completely resolved, with an NRS score of 0 and GERD-Q score of 1 (Table 1).

**TABLE 1:** Follow-up outcomes after phrenic nerve radiofrequency.

Assessment parameter	Pre-treatment	1 Month after SGBs and lidocaine infusion	1 Month Post-PNRF	2 Month Post-PNRF	3 Month Post-PNRF
Frequency of hiccup episodes (times/day)	>10	2–3	1–2	0–2	None
Duration of each episode	10–30 min	30 min	5–10 min	5 min	None
NRS hiccup severity score	7	4	2	1	0
GERD-Q score	12	10	6	3	1
Pregabalin (mg/day)	300	300	150	150	0
Olanzapine (mg/day)	2.5	2.5	0	0	0
Paroxetine (mg/day)	20	20	20	20	20

NRS, Hiccup Severity Score: A scale from 0 to 10, where 0 indicates no severity and 10 indicates the most severe intensity of hiccup episodes; GERD-Q, Score: A score used to evaluate GERD, symptoms, ranging from 0 to 18. Higher scores indicate more severe symptoms.

## Discussion

This case illustrates the potential efficacy of a dual-modality approach combining ultrasound-guided PNRF and SGBs for managing long-standing, intractable hiccups. The patient’s 20-year history of refractory hiccups suggests a complex interplay between peripheral and central mechanisms, with gastric irritation likely serving as an initial trigger and central sensitization amplifying the persistence of symptoms.

The central control of hiccups is mediated by a distinct reflex arc, which is separate from the autonomic respiratory centers. Key components of this circuit include the medulla oblongata, periaqueductal gray, subthalamic nuclei, phrenic nerve motor nucleus, the reticular formation, and hypothalamus (Nausheen et al., 2016). Clinical observations have linked structural or functional abnormalities in these regions to intractable hiccups, including cervical spinal cord lesions (Hao et al., 2013), thalamic or basal ganglia insults (Sweeney et al., 2018), and disruptions in neurotransmitter balance (Caloro et al., 2016). Imaging in this patient ruled out structural central pathology, supporting peripheral modulation as a therapeutic target. Noninvasive strategies are often recommended as first-line measures for acute hiccups. In the present case, however, the patient described episodes as continuous and intense, which limited the feasibility of performing vagal maneuvers; these techniques had been attempted previously without perceived benefit, and the lack of prodromal symptoms prevented their routine application during symptom-free periods. The phrenic nerve, originating from C3-C5 and innervating the diaphragm, constitutes the final efferent limb of the hiccup reflex arc. Interventions directed at this pathway have been shown to suppress diaphragmatic irritability and relieve intractable hiccups, although local anesthetic blocks often provide only transient benefit (Calvo et al., 2002). In this context, PNRF may achieve longer-lasting effects by modulating excitatory C-fiber activity and altering cytokine expression (Park et al., 2019), thereby extending clinical efficacy beyond that of simple nerve block (Kang et al., 2010).

The role of SGB in this case was adjunctive but mechanistically relevant. Although direct evidence linking sympathetic overactivity to hiccup initiation remains limited, SGB has been shown to modulate central autonomic circuits, reduce sympathetic outflow, and improve regional cerebral perfusion. Importantly, multiple studies demonstrate that SGB decreases pro-inflammatory cytokines and attenuates neuroinflammation (Lopez and Kumar, 2023; Sun et al., 2024; Kostadinov et al., 2025), which may contribute to reducing excitability along the hiccup reflex arc. Given that PNRF has also been associated with downregulation of local cytokine expression, the combined anti-inflammatory and autonomic-modulating effects of SGB may have created a favorable physiologic environment for sustained symptom control. Thus, the combination of PNRF and SGB was used pragmatically to address both somatic and autonomic components of the reflex arc.

Another important consideration is the role of IV lidocaine. As reported in several case studies (Coh et al., 2001; Dunst et al., 1993; Boulouffe and Vanpee, 2007), IV lidocaine can rapidly resolve persistent hiccups, likely through central desensitization and sodium-channel blockade. In our patient, IV lidocaine produced only transient improvement without meaningful functional recovery, suggesting that long-standing neuroplastic changes or phrenic nerve hyperexcitability may have limited its impact. This limited response reinforced the need for targeted neuromodulatory interventions. However, it is important to acknowledge that the multimodal nature of treatment, including pharmacologic therapy, phrenic nerve block, SGB, PNRF, and IV lidocaine, introduces overlapping therapeutic effects that cannot be completely disentangled.

We acknowledge important limitations. Because multiple interventions were applied in sequence, it is difficult to isolate the precise contribution of each procedure. This reflects the reality of managing highly refractory cases, where clinical urgency often necessitates pragmatic escalation rather than controlled evaluation of single modalities. Moreover, as a single case, our findings cannot be generalized; larger studies are needed to validate this combined strategy and clarify the optimal sequencing of interventions. It is also noteworthy that gastrointestinal treatments improved reflux symptoms and mucosal healing, but did not resolve hiccups, underscoring that chronic hiccups may persist independently of gastrointestinal pathology and require targeted neural modulation.

## Conclusion

This case supports the use of ultrasound-guided PNRF combined with SGBs as a viable therapeutic option for intractable hiccups. In the absence of structural central lesions, targeting the peripheral components of the hiccup reflex arc provides a logical and potentially effective treatment approach. The synergistic modulation of both somatic and sympathetic pathways may play a key role in symptom relief, particularly in cases unresponsive to conventional pharmacologic or local anesthetic interventions. However, further studies are needed to refine patient selection criteria and optimize treatment protocols.

## Data Availability

The raw data supporting the conclusions of this article will be made available by the authors, without undue reservation.
